# Enisamium Reduces Influenza Virus Shedding and Improves Patient Recovery by Inhibiting Viral RNA Polymerase Activity

**DOI:** 10.1128/AAC.02605-20

**Published:** 2021-03-18

**Authors:** Aartjan J. W. te Velthuis, Tatiana G. Zubkova, Megan Shaw, Andrew Mehle, David Boltz, Norbert Gmeinwieser, Holger Stammer, Jens Milde, Lutz Müller, Victor Margitich

**Affiliations:** a Smorodintsev Research Institute of Influenza, Ministry of Health of the Russian Federation, Saint Petersburg, Russia; b Division of Virology, Department of Pathology, Addenbrooke’s Hospital, University of Cambridge, Cambridge, United Kingdom; c Department of Microbiology, Icahn School of Medicine at Mount Sinai, New York, New York, USA; d Department of Medical Microbiology & Immunology, University of Wisconsin—Madison, Madison, Wisconsin, USA; e IIT Research Institute, Chicago, Illinois, USA; f Pharmalog Institut für klinische Forschung GmbH, Ismaning, Germany; g Dr. Regenold GmbH, Badenweiler, Germany; h Farmak JSC, Kyiv, Ukraine

**Keywords:** treatment, respiratory viruses, influenza A and B viruses, respiratory syncytial virus, adenovirus, enisamium iodide, FAV00A, RNA polymerase, RNA synthesis, adenoviruses, influenza A and B

## Abstract

Infections with respiratory viruses constitute a huge burden on our health and economy. Antivirals against some respiratory viruses are available, but further options are urgently needed. Enisamium iodide (laboratory code FAV00A, trade name Amizon) is an antiviral, marketed in countries of the Commonwealth of Independent States for the treatment of viral respiratory infections, but its clinical efficacy and mode of action are not well understood. In this study, we investigated the efficacy of enisamium in patients aged between 18 and 60 years with confirmed influenza virus and other viral respiratory infections. Enisamium treatment resulted in reduced influenza virus shedding (at day 3, 71.2% in the enisamium group tested negative versus 25.0% in placebo group [*P* < 0.0001]), faster patient recovery (at day 14, 93.9% in the enisamium group had recovered versus 32.5% in placebo group [*P* < 0.0001]), and reduced disease symptoms (from 9.6 ± 0.7 to 4.6 ± 0.9 score points in enisamium group versus 9.7 ± 1.1 to 5.6 ± 1.1 score points in placebo group [*P* < 0.0001]) compared to those in the placebo group. Using mass spectrometry, and cell-based and cell-free viral RNA synthesis assays, we identified a hydroxylated metabolite of enisamium, VR17-04. VR17-04 is capable of inhibiting influenza virus RNA synthesis and is present in plasma of patients treated with enisamium. VR17-04 inhibits the activity of the influenza virus RNA polymerase more potently than its parent compound. Overall, these results suggest that enisamium is metabolized in humans to an inhibitor of the influenza virus RNA polymerase that reduces viral shedding and improves patient recovery in influenza patients. (This study has been registered at ClinicalTrials.gov under identifier NCT04682444.)

## Abstract (precis)

Infections with respiratory viruses constitute a huge burden on our health and economy. Antivirals against some respiratory viruses are available, but further options are urgently needed. Enisamium iodide (laboratory code FAV00A, trade name Amizon) is an antiviral marketed in countries of the Commonwealth of Independent States for the treatment of viral respiratory infections, but its clinical efficacy and mode of action are not well understood.

## INTRODUCTION

Infections with respiratory viruses, such as influenza A virus (IAV), are the cause of morbidity and mortality in humans and a source of serious medical and socioeconomic problems for human health worldwide. Of additional concern is the simultaneous circulation of several respiratory viruses in the human population, including influenza B virus (IBV), adenoviruses (ADV), parainfluenza viruses (PIV), respiratory syncytial virus (RSV), coronaviruses (CoV), and various IAV subtypes (e.g., H3N2 and H1N1). All these viruses have the potential to spread rapidly among the human population, become resistant to antivirals, cause severe disease, and be associated with secondary complications (1). Moreover, novel respiratory viruses may emerge from animal reservoirs, such as wild birds or bats, and cause a pandemic when no or little preexisting immunity exists in the human population. Examples of recent outbreaks are the severe acute respiratory syndrome coronavirus 2 (SARS-CoV-2) pandemic virus in 2019 to 2021 and the H1N1 pandemic IAV strain in 2009 to 2010. Together, these characteristics make respiratory viruses a major threat to human health and extraordinarily difficult targets for the development of preventive and therapeutic mitigation measures.

Seasonal IAV and IBV infections can be prevented by inactivated or live attenuated vaccines. Vaccines have also been approved for emerging highly pathogenic IAV H5N1 or H7N9 strains and pandemic SARS-CoV-2. However, the efficacy of these vaccines is dependent upon antigenic similarity between the vaccine strain and the circulating viruses and is potentially diminished in high-risk groups (2, 3). Furthermore, the lead time to produce new vaccines is long. Currently, no vaccines exist against human seasonal CoV or RSV infections, although vaccines are in development (4–6).

Drug-based antiviral therapies are an important mitigation strategy against respiratory virus infections. In the case of IAV infections, drugs are available that target the viral neuraminidase protein (e.g., oseltamivir, zanamivir, peramivir, and laninamivir), the IAV RNA-dependent RNA polymerase (e.g., favipiravir [T-705]), or the endonuclease domain of polymerase acidic (PA) protein (e.g., baloxavir marboxil) (7, 8). The emergence of resistant viruses has been reported for most of these antivirals (9, 10), and research into alternative antiviral therapies with a different mode of action is needed with high priority.

Enisamium is an isonicotinic acid derivative. The iodide salt of enisamium (lab code FAV00A), formerly named carbabenzpyride, is marketed as Amizon in 11 countries, including in former Soviet Union countries and Mongolia, for the prophylaxis and treatment of influenza and other viral diseases, both in adults and children (11). Previous studies indicate that enisamium inhibits several IAV strains, including A/Brisbane/59/2007 (H1N1), A/Tennessee/1-560/2009 (H1N1), A/Perth/16/2009 (H3N2), A/Vietnam/1203/2004 (H5N1), and A/Anhui/1/2013 (H7N9), and the replication of IBV in cell culture (12).

IAVs and IBV are negative-sense RNA viruses whose 14-kb genome consists of eight segments of single-stranded viral RNA (vRNA). The viral RNA-dependent RNA polymerase copies the vRNA into a replicative intermediate called the cRNA during viral replication or into capped and polyadenylated viral mRNA (mRNA) during viral transcription (13). The cRNA serves as a template for the production of new vRNA molecules. vRNA and cRNA molecules are both replicated in the context of ribonucleoproteins (RNPs), which consist of a copy of the RNA polymerase complex bound to the 5′ and 3′ ends of a genome segment and a helical coil of nucleoproteins (NPs) that are bound to the rest of the vRNA or cRNA (13). The IAV and IBV RNA-dependent RNA polymerases are composed of three subunits: polymerase basic 1 (PB1) protein, PB2 protein, and PA protein. All three protein subunits are essential for replication and transcription of the viral RNA genome segments (13).

Here, we present a comprehensive analysis of the efficacy of enisamium against IAV *in vitro* and IAV and IBV infections in patients. We found that enisamium is effective in reducing viral shedding and improving patient recovery in influenza virus-infected patients. Subsequent mass spectroscopy analysis of sera from enisamium-treated patients revealed the presence of a hydroxylated metabolite of enisamium, VR17-04. Using cell-free RNA polymerase activity experiments, we determined that the metabolite VR17-04 is a more potent inhibitor of influenza A viral RNA synthesis than enisamium. Overall, these results suggest that enisamium is effective in reducing virus shedding and improving recovery of influenza patients, and that its mode of action is inhibition of the influenza virus RNA polymerase, most likely via the active metabolite VR17-04.

## RESULTS

### Patient cohort was predominantly infected with influenza A and B viruses.

**FIG 1:**
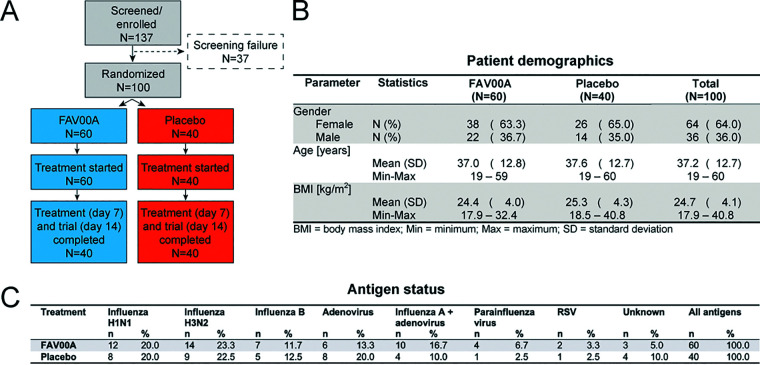
Patient enrollment, demographics, and antigen status. (A) Schematic of patient recruitment, randomization, and treatment. (B) Demographics of enisamium- and placebo-treated patient groups. (C) Frequency of virus antigen detection in nasal swabs of patients treated with enisamium or placebo by immunofluorescence staining.

A total of 137 patients were recruited and screened for respiratory virus infections, and 100 were randomized for treatment with enisamium (*n* = 60) or the placebo control (*n* = 40) (Fig. 1A). Age and gender distributions were similar between the two groups (Fig. 1B). All patients completed the trial as per protocol, and no dropouts occurred. At the start of the clinical trial, the patients reported 1.6 ± 0.5 (mean ± standard deviation [SD]) days without routine activity due to respiratory infection. Antigen testing showed that the patients treated with enisamium or placebo were positive for IAV (43.0%), ADV (16.0%), a combination of IAV and ADV (14.0%), or IBV (12.0%). An additional 8.0% of patients tested positive for PIV or RSV, whereas 7% of the patients (5% in the enisamium group and 10% in the placebo group) tested negative for IAV, IBV, ADV, PIV, or RSV antigen. Overall, 69% of patients were infected with influenza A and/or B viruses. The enisamium- and placebo-treated groups had similar distributions of respiratory viruses (Fig. 1C). Throughout the study, there was no deviation from the scheduled medication intake, with a mean treatment duration of 6.8 ± 0.8 days. Topical decongestants were used for a shorter duration of treatment and by a lower proportion of patients treated with enisamium than for the placebo group (23.3% [*n* = 14] versus 75% [*n* = 30]). Expectorants were used by all patients in both treatment groups, but the majority of the enisamium-treated patients used the drug for 7 days, compared to 14 days for the placebo-treated patients.

### Enisamium treatment reduces viral antigen levels and improves activity in patients with viral respiratory disease.

**FIG 2:**
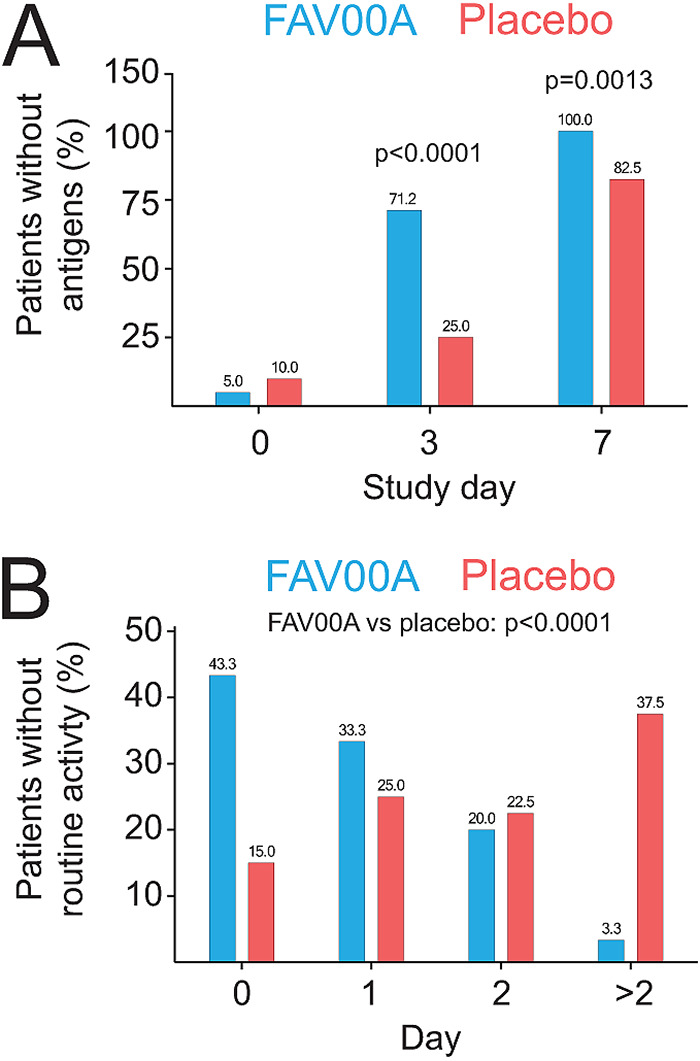
Enisamium treatment reduces viral antigen levels and improves patient activity. (A) Patients in whom virus antigens were not detected by immunofluorescence staining of nasal swabs. (B) Patients without routine activities. *P* values were determined by Fisher’s exact test.

Antigen testing on day 3, the first visit after initiation of treatment, showed that 71.2% (*n* = 42) of the patients treated with enisamium and 25.0% (*n* = 10) of the placebo group were negative for the above-mentioned respiratory virus antigens (*P* < 0.0001). On day 7, the second visit after initiation of treatment, all patients treated with enisamium tested negative for respiratory virus antigens, while 82.5% (*n* = 33) of the placebo control patients tested negative (*P* = 0.0013) (Fig. 2A). The proportion of patients that tested antigen negative on days 3 and 7 after initiation of treatment was significantly higher (*P* < 0.0001) in the enisamium-treated group (70.7% [*n* = 41]) than in the placebo-treated group (25.0% [*n* = 10]). A subgroup analysis indicated a similar positive effect of the enisamium treatment compared to the placebo for patients infected with IAV or IBV (75.0% [*n* = 24] and 22.7% [*n* = 5], respectively [see Fig. S1 in the supplemental material]) or ADV (71.4% [*n* = 5] and 12.5% [*n* = 1], respectively). Analysis of patient behavior during the clinical study showed that the proportion of patients with no or just 1 day without routine activities after the start of the treatment was higher in the enisamium-treated group than in the placebo group. In the latter group, the majority of patients was not able to perform routine activities for two or more days (Fig. 2B). These group differences were highly significant (*P* < 0.0001).

### Enisamium treatment reduces objective symptoms in patients with viral respiratory disease.

**FIG 3:**
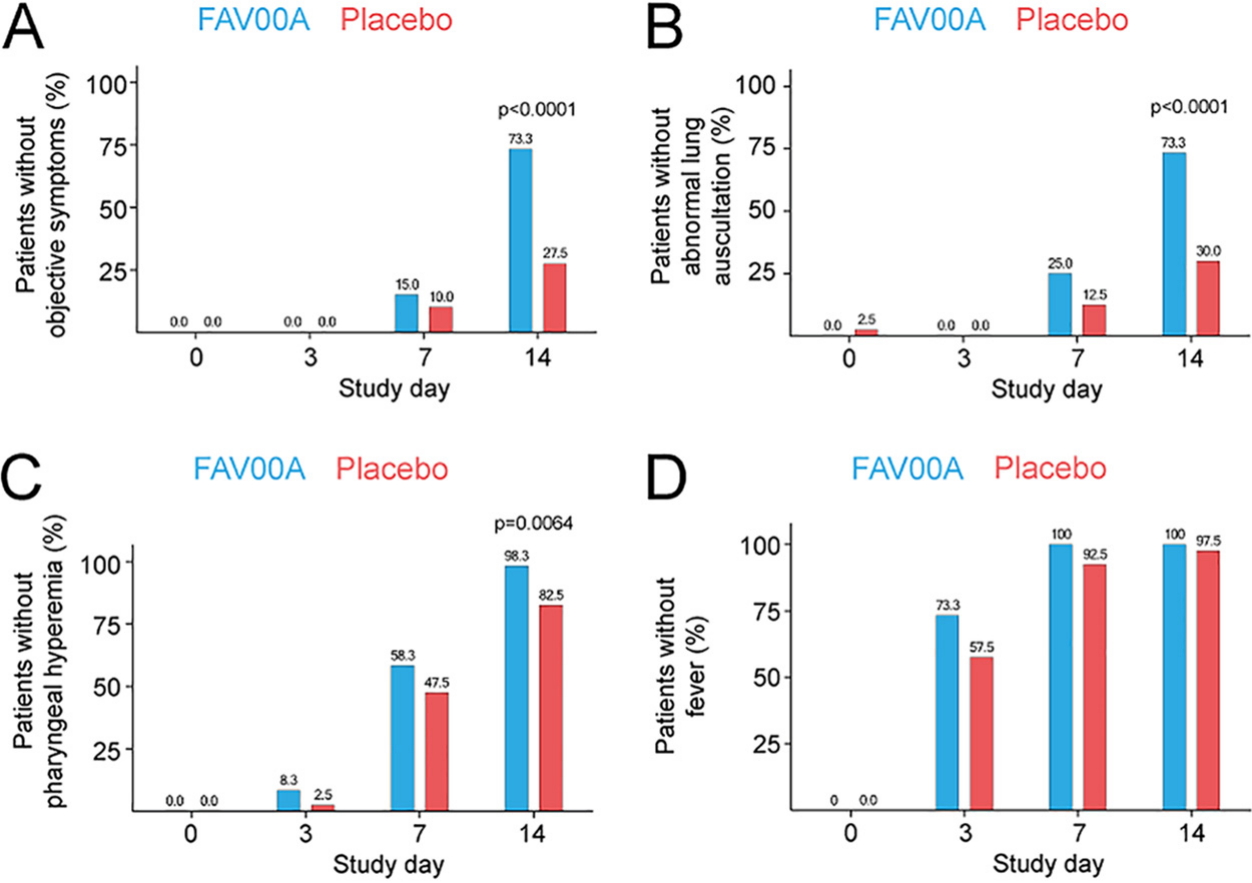
Enisamium treatment reduces objective symptoms in patients with viral respiratory disease. (A) Patients without objective symptoms at different visit days. (B) Patients without abnormal breath sounds at different visit days. (C) Patients without pharyngeal hyperemia at different visit days. (D) Patients without fever at different visit days. *P* values were determined by Fisher’s exact test.

Analysis of the objective symptoms fever, pharyngeal hyperemia, and abnormal lung auscultation contributed mainly to the objective symptom score at the start of the clinical trial on day 0. There was no difference between the objective symptom scores between the two groups on day 0 (9.6 ± 0.7 for the enisamium group and 9.7 ± 1.1 for the placebo group). A low proportion of trial participants presented with conjunctival infection (22.0%) and enlarged lymph nodes (1.0%), and none had abnormal auscultation of the heart or arterial blood pressure at enrollment. The decrease in the objective symptom score from day 0 until day 14 was statistically significant (*P* < 0.0001) in the enisamium-treated group (from 9.6 ± 0.7 to 4.6 ± 0.9 score points) compared to the placebo group (from 9.7 ± 1.1 to 5.6 ± 1.1 score points). An absence of all objective symptoms was observed in both patient groups starting from day 7 (Fig. 3A), and a significant increase in the absence of all 3 symptoms was observed in the enisamium-treated patients on day 14 (Fig. 3A; *P* < 0.0001). Fever, pharyngeal hyperemia, and lung auscultation were similar in both treatment groups on day 0, and pharyngeal hyperemia as well as lung auscultation resolved significantly faster in the enisamium group than in the placebo group (Fig. 3B and C). Similar results were obtained for the subgroup of patients suffering from influenza virus infection only (Fig. S2A to D). Together, these observations strongly suggest that enisamium treatment leads to a faster and highly significant reduction in objective clinical symptoms associated with respiratory virus infections.

### Enisamium treatment reduces subjective symptoms in patients with viral respiratory disease.

The subjective symptoms weakness, headache, increased perceived body temperature, sore throat, and cough were present in all patients on day 0 and contributed most to the mean subjective symptom score. Less than half of the patients reported myalgia (46.0% [*n* = 46]) and just a few participants had chills (7.0% [*n* = 7]) at day 0. However, the subjective symptoms were predominantly of mild intensity and almost absent on day 3 in both groups, meaning that they had limited impact on the score.

**FIG 4:**
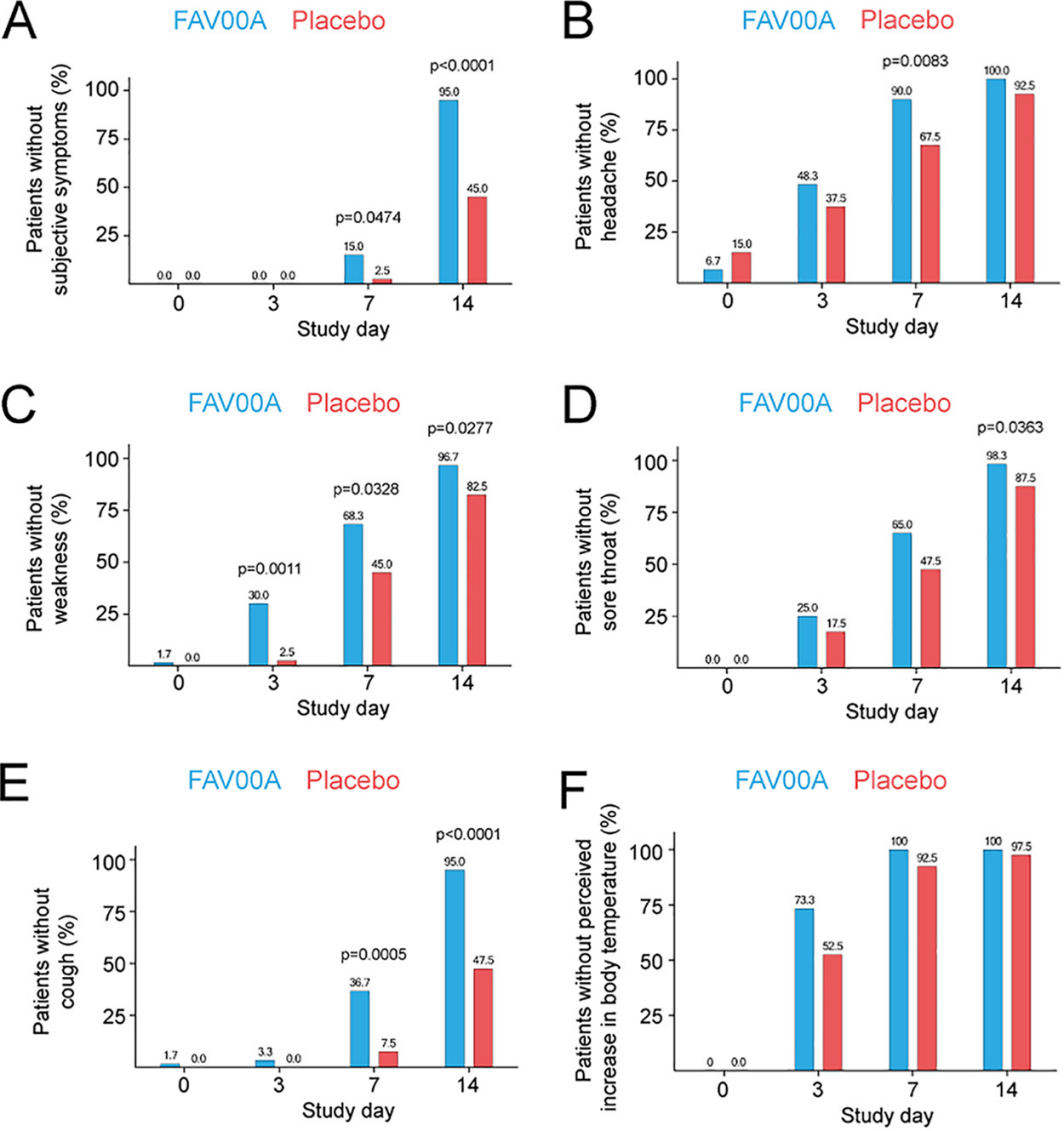
Enisamium treatment reduces subjective symptoms in patients with viral respiratory disease. (A) Patients without subjective symptoms at different visit days. (B) Patients without headache at different visit days. (C) Patients without weakness at different visit days. (D) Patients without sore throat at different visit days. (E) Patients without cough at different visit days. (F) Patients without elevated body temperature at different visit days. *P* values were determined by Fisher’s exact test.

Analysis of the progression of subjective symptoms over time revealed a more pronounced decrease in subjective symptoms in the enisamium-treated group compared to the placebo group at days 3, 7, and 14 after initiation of treatment (Fig. 4). Specifically, we found that by day 14, the score had decreased from 15.7 ± 2.2 to 7.1 ± 0.5 in the enisamium group, compared to 15.4 ± 1.8 to 8.0 ± 1.3 in the placebo group. The mean group differences were −1.2 score points on day 7 (*P* < 0.0001) and −0.9 score point each on day 3 (*P* = 0.0065) and day 14 (*P* < 0.0001). In addition, we found that the absence of all above-mentioned subjective symptoms was more pronounced in the enisamium-treated group on both day 7 and day 14 compared to the placebo control (Fig. 4A). We also found that the subjective symptoms that contributed most to the sum score (i.e., weakness, headache, increased perceived body temperature, sore throat, and cough) abated faster in the enisamium-treated group than in the placebo group (Fig. 4B to F). These results were also found to be true when treated and nontreated patients suffering from different virus infections were compared on day 14 (for influenza virus subgroup, see Fig. S3A to F). These findings suggest that enisamium treatment has a positive outcome for clinically relevant subjective symptoms, such as feelings of weakness, headache, sore throat, and coughing.

### Enisamium treatment leads to faster patient recovery.

Next, we analyzed the recovery time of enisamium-treated and placebo-treated patients in terms of resolution of symptoms and return to premorbid health status. In support of the above-described observations, a complete recovery from symptoms associated with the viral respiratory infections was reported by 18.3% (*n* = 11) of the patients treated with enisamium on day 7, while none in the placebo group had fully recovered on that day. At day 14, 93.3% (*n* = 56) of the FAV00A-treated patients had fully recovered, whereas only 32.5% (*n* = 13) of patients in the placebo group returned to normal health status. A significant improvement in the patient’s health status was reported by 60.0% (*n* = 36) of the enisamium-treated patients, compared to 15.0% (*n* = 6) of the placebo-treated patients, on day 3 (*P* < 0.0001). FAV00A treatment led to a significant improvement in patient health at all three visit days after initiation of treatment (days 3, 7, and 14 [*P* < 0.0001 to *P* = 0.0018]) compared to that in the placebo group.

While enisamium treatment generally led to faster patient recovery, 11 adverse events (AEs) occurred during the clinical trial. Of these, 4 AEs were reported by 4 patients (6.7%) treated with enisamium (bitter taste in the mouth in 2 patients and heartburn and burning sensation in the throat in 1 patient each). The other 7 AEs in the enisamium-treated patients were of mild intensity and resolved without additional therapy. Gastrointestinal side effects as reported by 4 patients are mentioned in the Amizon drug product label (summary of product characteristics), which suggests that the AEs were related to Amizon.

### Enisamium inhibits influenza A virus infection in cell culture.

**FIG 5:**
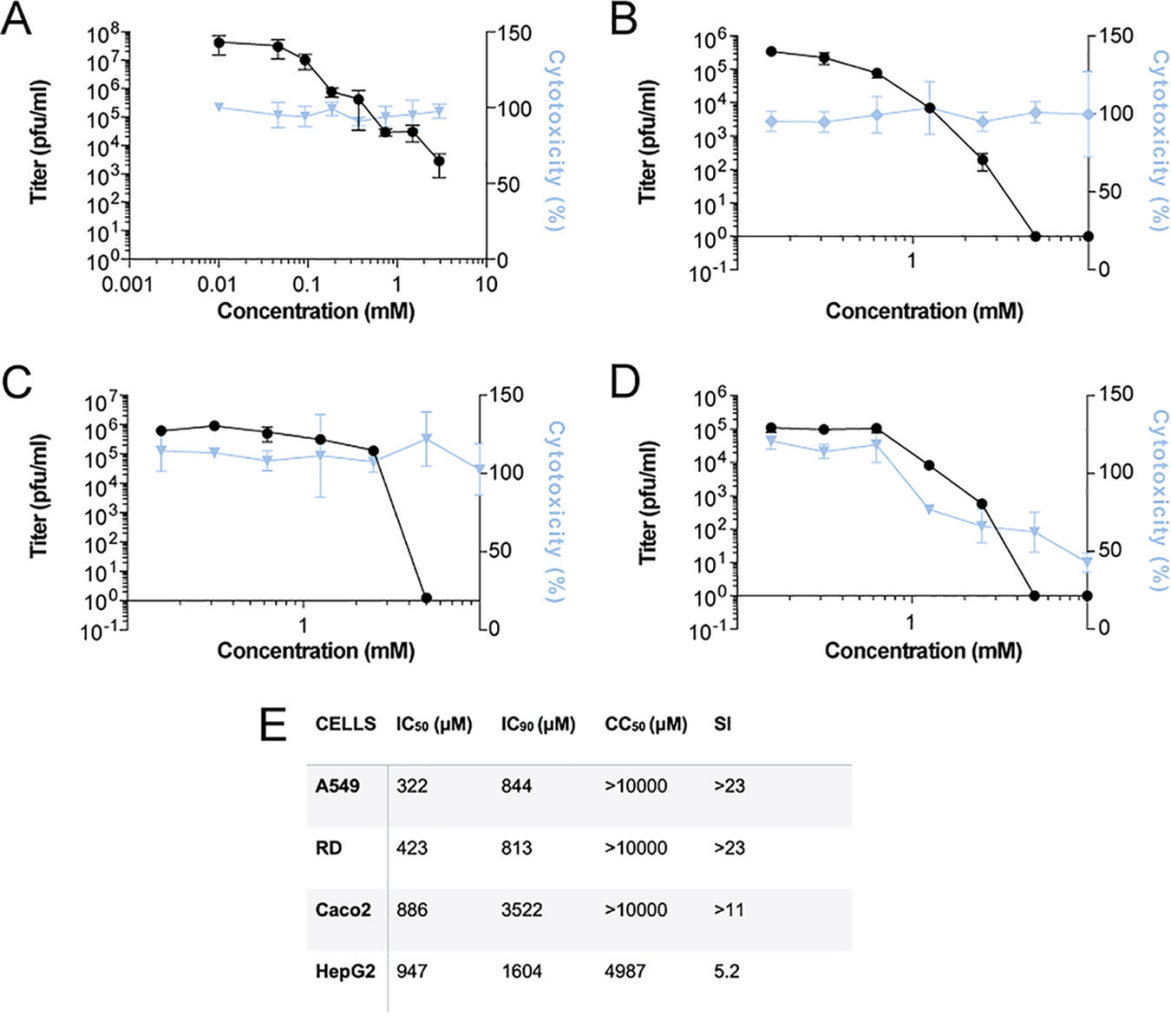
Inhibition of IAV infection and RNA synthesis by enisamium in cell culture. (A) Effect of enisamium on influenza A/WSN/1933 (H1N1) virus titers in A549 cells as determined by plaque assay (black line). Cytotoxicity (blue line) was determined in uninfected cells after 48 h of incubation with FAV00A. (B to D) Effect of enisamium on influenza A/WSN/1933 (H1N1) virus titers in RD cells (B), Caco-2 cells (C), and HepG2 cells (D) as determined by microplaque assay (black line). Cytotoxicity (percentage of live cells, blue line) was determined in uninfected cells after 48 h of incubation with enisamium. Data points represent means and standard deviations of three independent enisamium titrations and matching virus plaque experiments. (E) Overview of IC_50_, IC_90_, 50% cytotoxic concentration (CC_50_), and selectivity index (SI) values in different cell lines.

Previous *in vitro* experiments showed that enisamium can inhibit IAV infection in normal human bronchial epithelial (NHBE) cultures (12). To confirm and extend these results, we incubated several human cell lines (A549, RD, Caco-2, and HepG2) with enisamium and subsequently infected these cell lines with influenza A/WSN/1933 (H1N1) virus (referred to as WSN) using multiplicities of infection (MOIs) that were optimized for each cell line. We found that enisamium significantly affected WSN titers in A549 cells, with a 50% inhibitory concentration (IC_50_) of 322 μM, in line with previous observations (Fig. 5A and E). Higher IC_50_ values were observed for the RD, Caco-2, and HepG2 cells (Fig. 5B to E). Selectivity indexes for enisamium were >11 for A549, RD, and Caco-2 cells (Fig. 5E) but lower for HepG2 cells due enisamium’s higher cytotoxicity in these cells (Fig. 5D and E). These data confirm that enisamium can inhibit influenza A virus infections in a variety of human cell lines derived from lung, liver, colon, and skeletal muscle tissues; however, its antiviral effects are strongly cell type dependent, with respiratory tract-derived cells such as NHBE (12) and A549 cells (Fig. 5A) being the best target cells for the anti-influenza A inhibitory activity of enisamium.

To test if influenza A viruses can develop resistance to enisamium, we passaged influenza A/Brisbane/59/2007 (H1N1) virus 10 times at an MOI of 0.005 in NHBE cells, after an initial infection at an MOI of 0.01. The concentration of enisamium chloride (125, 250, and 500 μg/ml) was kept constant through the passaging experiment, and viruses were allowed to grow for 48 h on the NHBE cells between passages 2 and 10. After each passage, the sensitivity of the isolated virus was tested in an enisamium titration experiment. No differences in sensitivity were observed after 10 passages (Fig. S4), suggesting that influenza A viruses do not quickly become resistant to enisamium.

### Enisamium inhibits IAV RNA polymerase activity in cell culture.

**FIG 6:**
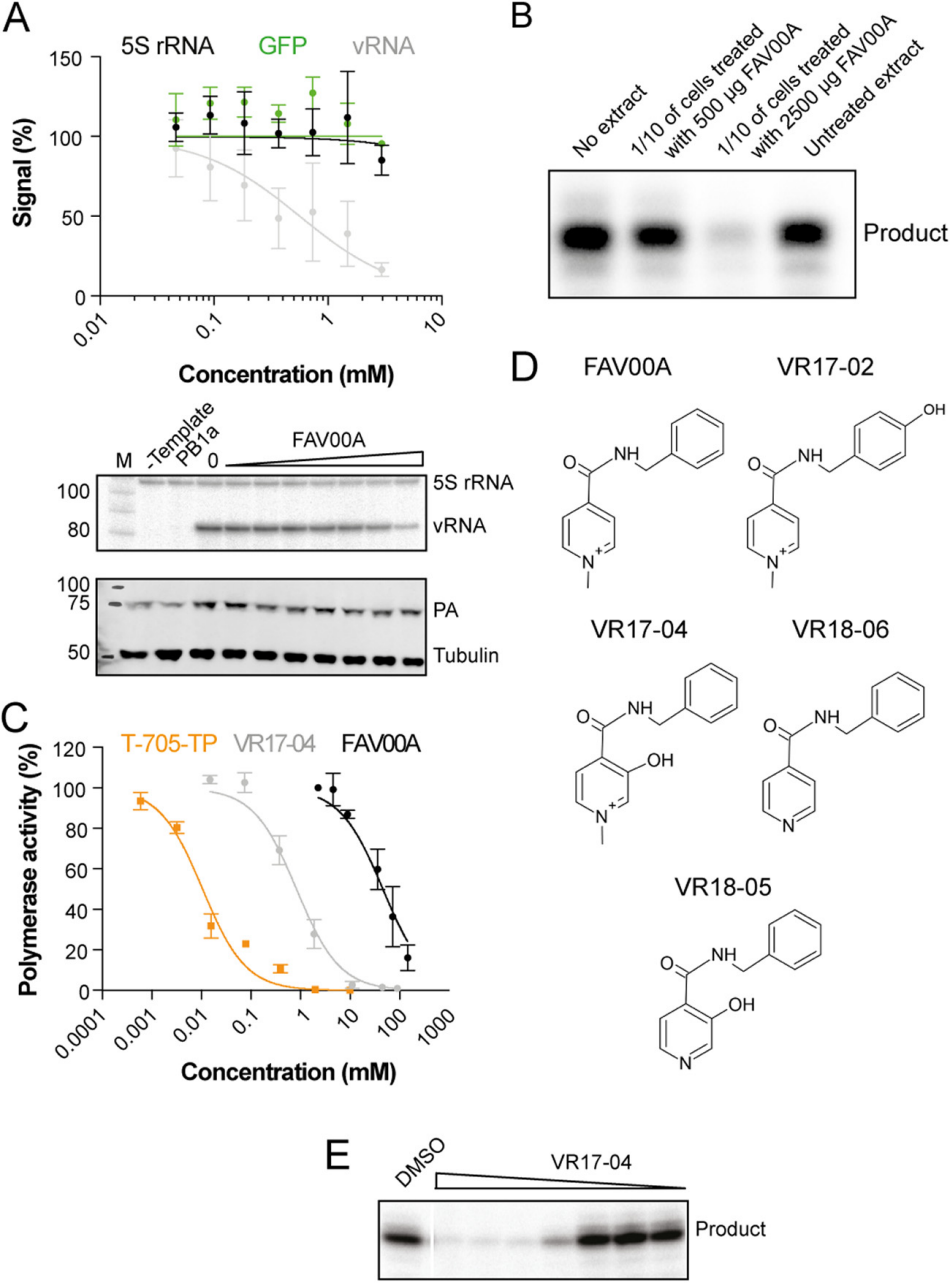
Enisamium is metabolized in humans and metabolite VR17-04 inhibits the viral RNA polymerase *in vitro*. (A) Effect of enisamium on the steady-state IAV vRNA, 5S rRNA, and GFP levels, with quantification shown in the graph. Levels of 5S rRNA and IAV vRNA were analyzed by primer extension (middle panel). PA and tubulin expression were analyzed by Western blotting (bottom panel). A mutant IAV RNA polymerase containing two amino acid substitutions in the PB1 active site (PB1a) was used as a negative control. Data points represent means and standard deviations of three independent enisamium titrations and matching GFP measurements or primer extensions. M, marker. (B) Effect of extracts from A549 cells treated with enisamium on IAV RNA polymerase activity *in vitro*. Five hundred or 2,500 μg of enisamium was added to A549 cells for 24 h. Next, cells were lysed and 1/10 of the lysate was added to *in vitro* polymerase assays. RNA polymerase products were analyzed by 20% denaturing PAGE. (C) Quantification of the activity of the IAV RNA polymerase *in vitro* in the presence of enisamium, VR17-04, or T-705 triphosphate (T-705-TP). Data points represent means and standard deviations of three independent titrations in RNA polymerase assays. (D) Phase I metabolites identified in human plasma samples. (E) Activity of the IAV RNA polymerase in the presence of different VR17-04 concentrations.

To test the mechanism of action for enisamium, experiments were designed to study its activity on IAV RNA synthesis. For this purpose, an influenza virus minigenome assay was used as follows. HEK 293T cells were transfected with plasmids expressing the IAV RNA polymerase subunits PB1, PB2, and PA (pcDNA3-PB1, pcDNA3-PB2, and pcDNA3-PA), the viral NP (pcDNA3-NP), and a vRNA template based on segment 5 (NP-encoding genome segment), all derived from the H1N1 WSN influenza virus (14, 15). After transfection, enisamium was added to the cell culture medium at the concentrations indicated in Fig. 6A. Total cellular RNA was extracted 24 h posttransfection, and IAV replication (vRNA synthesis) activity was determined by radioactive primer extension analysis (15). Enisamium treatment significantly reduced the replication capacity of the viral RNA polymerase, with an IC_50_ of 354 μM. To estimate the effect of enisamium on host cell RNA synthesis, we transfected a plasmid expressing enhanced green fluorescent protein (eGFP) from a constitutively active cytomegalovirus (CMV) promoter and measured eGFP expression in the cell suspension of enisamium-treated cell cultures using GFP fluorescence. As a further control, we analyzed the effect of enisamium treatment on the rRNA steady-state level. No significant effect on 5S rRNA or GFP expression levels was observed in the presence of even higher concentrations (>2 mM) of FAV00A (Fig. 6A). Overall, these observations suggest that enisamium directly affects influenza viral RNA polymerase activity in cell-based assays.

### Enisamium weakly inhibits IAV RNA polymerase activity *in vitro*.

To examine the effect of enisamium on IAV RNA synthesis *in vitro*, we expressed protein A-tagged RNA polymerase from A/WSN/1933 (H1N1) virus in HEK 293T cells and purified the heterotrimeric tandem affinity protein (TAP)-tagged polymerase complex using IgG Sepharose chromatography. We then performed *in vitro* RNA synthesis assays using a model 14-nucleotide (nt) vRNA template in the presence of different concentrations of enisamium, or T-705 triphosphate (Fig. 6B) as a positive control. In these assays, enisamium weakly inhibited IAV RNA polymerase activity, with an IC_50_ of 46.3 mM, while T-705 triphosphate efficiently inhibited the RNA polymerase, with a mean IC_50_ of 0.011 mM (Fig. 6B). This suggests that enisamium exerts an inhibitory effect on the IAV RNA polymerase activity *in vitro*, although its inhibitory activity is rather limited.

### Enisamium is metabolized in humans and metabolite VR17-04 inhibits IAV RNA polymerase activity.

The weak inhibition of the IAV RNA polymerase activity by enisamium is unlikely to be responsible for the obvious antiviral efficacy of enisamium observed in cell culture and in patients. Based on the data obtained with enisamium treatment in different cell types (Fig. 5), we hypothesized that a metabolite of enisamium could be the actual inhibitor of the IAV RNA polymerase in cell culture. To test this hypothesis, we treated A549 cells with enisamium for 24 h, lysed the cells, and then performed IAV RNA polymerase activity assays in the presence of the cellular extract. We observed a strong, concentration-dependent inhibition of IAV RNA polymerase activity, which is consistent with the hypothesis that a metabolite of enisamium inhibits influenza virus RNA synthesis during IAV infection (Fig. 6C).

To confirm that enisamium is metabolized in humans, we analyzed human plasma samples from a phase I pharmacokinetic study using high-performance liquid chromatography (HPLC) and tandem mass spectrometry (MS/MS). This analysis revealed the presence of four phase I metabolites (Fig. 6D), of which two were hydroxylated (VR17-02 and VR17-04), one was demethylated (VR18-06), and one was both hydroxylated and demethylated (VR18-05). We also detected phase II metabolites that had been formed by glucuronidation or sulfate conjugation of phase I metabolites. Since glucuronidation or sulfate conjugation typically result in drug inactivation, we did not study the phase II metabolites further. In order to test if enisamium phase I metabolites have a direct effect on IAV RNA polymerase activity *in vitro*, the phase I metabolites were synthesized. Only VR17-04 was sufficiently soluble in dimethyl sulfoxide (DMSO) for *in vitro* testing and subsequently tested in *in vitro* IAV RNA polymerase assays containing purified IAV RNA polymerase, a 14-nt-long vRNA, ApG, radiolabeled [α-^32^P]GTP, and all four unlabeled nucleoside triphosphates (NTPs) (14). Analysis of the reactions by 20% denaturing PAGE revealed a strong inhibition of viral RNA synthesis in the presence of the hydroxylated metabolite VR17-04 compared to the DMSO control (Fig. 6E). Quantitation of the reactions showed that the human metabolite VR17-04 inhibited IAV RNA polymerase activity 55-fold more strongly (IC_50_ of 0.84 mM) than enisamium (IC_50_ of 46 mM) (Fig. 6C).

## DISCUSSION

Infections with respiratory viruses create a huge burden on our health and economy, but currently, only a limited number of antivirals are available against the viruses that cause them. In this study, we investigated the efficacy and safety of enisamium in patients aged between 18 and 60 years with clinically and virologically confirmed influenza virus and other viral respiratory infections. The clinical study demonstrated that treatment with enisamium resulted in reduced virus shedding and a reduced mean number of days without routine activities in patients with viral respiratory infections, compared to those in the placebo group. In addition, objective symptoms (fever and pharyngeal hyperemia) and subjective symptoms (weakness, headache, increased perceived body temperature, sore throat, and cough) were reduced and disappeared more quickly in patients receiving enisamium treatment within 24 h of the onset of symptoms. These results suggest (i) a direct clinical effect of enisamium on the outcome of viral respiratory infections in humans and (ii) that enisamium treatment leads to faster patient recovery and a reduction of symptoms.

The use of concomitant symptomatic therapy (e.g., topical decongestants and expectorants) as well as the impact of the concomitant medication on the evaluation of the symptoms could affect clinical trial results. However, in the enisamium-treated group, symptomatic drugs were taken less frequently and for shorter durations than in the placebo group, suggesting that an overestimation of the enisamium effects due to concomitant symptomatic therapy can be excluded. In fact, it is tempting to suggest that enisamium treatment led to reduced symptomatic therapy in the enisamium-treated group. Moreover, in recent clinical trials with influenza virus neuraminidase inhibitors (e.g., oseltamivir), the evaluated symptoms, such as fever, chills, headache, muscle ache, and cough, as well as other clinical parameters, such as return to routine activities, were similar to the symptoms and parameters evaluated in this clinical trial (16, 17). Therefore, the methods used to evaluate the efficacy of enisamium for human respiratory virus infections and the results presented here should be reliable.

We also evaluated symptoms that are not characteristic for respiratory tract infections, such as abnormal arterial blood pressure, abnormal auscultation findings of the heart, enlarged lymph nodes, and conjunctival infection. Most of these symptoms were indeed absent in the trial population or present in only a low percentage of the patients and of mild intensity. These noncharacteristic symptoms were revealed in all or almost all patients on day 3 after initiation of treatment in both treatment groups, suggesting that these atypical symptoms did not contribute considerably to the outcome of the clinical trial. The patients included in this study had primarily mild to moderate respiratory disease. Thus, the efficacy of enisamium against severe forms of respiratory disease or for high-risk groups of patients needs to be confirmed. The results will also need to be confirmed in a double-blinded clinical trial, since the current study relied on blinded patients but unblinded investigators. While our study included predominantly patients infected with influenza viruses or coinfections with influenza viruses and adenovirus, the results suggest that enisamium can be effective against other respiratory virus infections. Indeed, results from other recent studies showed that enisamium inhibits SARS-CoV-2 and adenovirus replication *in vitro* (18, 19).

Analysis of serum samples from enisamium-treated patients revealed a number of enisamium metabolites, suggesting that not enisamium but one or more metabolites may inhibit respiratory virus infections. A previous study had suggested that enisamium inhibits the RNA synthesis of various influenza A virus strains (12). In this study, we confirmed the inhibitory effect of enisamium on influenza virus RNA synthesis by employing influenza virus minigenome experiments (Fig. 6). Moreover, using *in vitro* influenza virus RNA polymerase assays, we demonstrated that enisamium can directly inhibit influenza virus replication and transcription and that one of its metabolites, VR17-04, is a more potent inhibitor of influenza virus RNA synthesis than enisamium (Fig. 6). These findings are in line with the inhibitory effect of extracts from cultured human lung cells treated with enisamium (Fig. 5). Using docking and molecular dynamics studies, we recently showed that VR17-04 can putatively base-pair with cytosine and adenosine bases in the RNA template when the template resides in the active site of the SARS-CoV-2 RNA polymerase, thereby preventing GTP and UTP incorporation (20). It is tempting to speculate that VR17-04 can inhibit the influenza A virus RNA polymerase in a similar manner and bind to cytosine and adenosine residues in the PB1 active site. We plan to address this hypothesis with detailed biochemical experiments in the future.

The IC_50_ of VR17-04 was substantially lower than the IC_50_ of favipiravir triphosphate (T-705-TP), our control in the *in vitro* experiments. However, our phase III clinical trial results suggest that enisamium may be more effective than favipiravir in uncomplicated influenza virus infections. Favipiravir triphosphate is taken up by cells as the prodrug T-705 and subsequently ribosylated and phosphorylated to form a triphosphorylated nucleoside analog (21). Pharmacokinetics studies with mice showed that T-705 quickly accumulates in the liver and is excreted by the kidney, indicating that only small amounts of T-705 accumulate in the respiratory tract (22). We have seen in preliminary experiments with rats that enisamium is highly enriched in the trachea (unpublished results); it is thus tempting to speculate that this distribution makes the antiviral effect of enisamium stronger than that of favipiravir in influenza patients.

In summary, we have performed a single-blinded clinical trial that shows that enisamium treatment of patients with viral respiratory infections leads to faster patient recovery and reduced virus shedding compared to those for the placebo control. Our results are supported by virus infections and viral replication *in vitro* studies on cells infected with IAV and with influenza viral RNA replication assays that suggest that enisamium can directly inhibit influenza viral replication through a hydroxylated metabolite, VR17-04. This study thus advances our understanding of enisamium and suggests that it could represent an alternative or additional treatment for current and emerging respiratory virus infections.

## MATERIALS AND METHODS

### Ethics of clinical study.

To investigate the efficacy and tolerability of enisamium in patients infected with respiratory viruses, a prospective, single-blinded, placebo-controlled, single-center clinical trial was performed. The study was conducted in accordance with the Declaration of Helsinki, International Council for Harmonization - Good Clinical Practice (ICH-GCP), and the national laws and regulations in the Research Institute of Influenza of the North-West Branch of the Russian Academy of Medical Sciences (NWB RAMS), St. Petersburg, Russian Federation, between 2009 and 2010. The study was approved by the Ethics Commission of the Research Institute of Influenza of the NWB RAMS (extract of report no. 28 of 9 April 2009) after review of the clinical trial protocol, investigator’s brochure, patient information with informed consent, case report forms, insurance policy, and the qualifications of the investigator. Patients provided written informed consent prior to study participation.

### Sample size of clinical study.

A sample size calculation was performed to design the clinical trial. The maximal difference between pre- and posttreatment parameters that was considered not significant was set to 0.25. Thus, a sample size of 30 patients in both the enisamium- and placebo-treated groups would be needed to demonstrate an efficacy of enisamium with a statistical power of 90% (bilateral) and a confidence level of 5%. We further estimated that about 15% of the patients would withdraw or be withdrawn (see exclusion criteria below) from the study prior to its completion. Using Fisher’s exact test for subsequent analyses, we estimated that a starting cohort of 50 patients per group would be sufficient to achieve the statistical power stated above.

### Patient cohort and treatment during the clinical study.

Male and female patients aged between 18 and 60 years with viral respiratory infections were eligible for participation in the clinical study. The diagnosis of viral respiratory infection was based on an axillary body temperature of ≥37.2°C, the presence of at least one symptom of respiratory tract infection (rhinitis, pharyngitis, laryngitis, tracheitis, bronchitis, or cough), and one general infection symptom (weakness, malaise, myalgia, headache, fever, or decreased appetite). In addition, diagnosis was confirmed by viral antigen immunofluorescence testing of nasal swabs. Patients with any acute organ dysfunction, serious chronic disease, pregnancy, or breastfeeding obligations were excluded from clinical trial participation. Patients were recruited by advertisements, randomized into two groups, and treated with either enisamium iodide (500 mg three times daily) or a matching placebo for 7 days. Treatment was started within 24 h of the onset of symptoms. Daily dosage and duration of treatment were in accordance with the summary of product characteristics of enisamium. The use of immunomodulatory drugs, systemic sympathomimetics, antipyretics, analgesics, and antibiotics was prohibited during the clinical trial, unless it was required for intervention. Endpoints of the study were the dynamics of clinical signs determined by investigating the disappearance of signs of rhinitis and the decrease of body temperature, interferon status, and reduction of the time to recovery.

### Data collection in clinical study.

During the course of the clinical trial, four visits were scheduled for each patient: a baseline visit (day 0) and three follow-up visits 3, 7, and 14 days thereafter. Objective disease symptoms were assessed by the investigator and subjective symptoms by the patients during each visit. The objective symptoms comprised fever (i.e., body temperature of ≥37.2°C), pharyngeal hyperemia, conjunctival infection, enlarged lymph nodes, abnormal arterial blood pressure, and auscultation findings (lung and heart). The subjective symptoms included weakness, myalgia, headache, increased body temperature (i.e., subjective severity rating), chills, sore throat, and cough. All patients were monitored for adverse events throughout the course of the clinical trial.

A score system was used to assess patient health. Objective symptoms were scored as follows: normal or abnormal blood pressure was counted 0 or 4 score points; lung auscultation was counted 0 for vesicular breath sound, and wheezing or crepitation were scored 2 or 4 points, respectively; and clear and rhythmic heart sounds were each scored 0 points, whereas noisy and arrhythmic heart sounds were scored 2 points each. The subjective symptoms were assessed using a 4-point Likert scale, ranging from 1 (absent) to 4 (severe). After patient assessment, a sum score was calculated. These scores ranged from 4 to 28 (minimum/maximum) for objective symptoms and from 7 to 28 score point for subjective symptoms.

In addition to scoring patient health, information about the number of days since day 0 without routine activities due to respiratory infection and overall treatment efficacy (complete, significant or moderate improvement, no significant change, or worsened) was recorded by the patient and investigator. Finally, nasal swabs were collected from the patients on days 0, 3, and 7 to determine the presence of viral antigens by viral antigen immunofluorescence testing. Viral titers were not determined.

### Statistical analysis.

Descriptive summary statistics were used for analyses of the clinical results. Patients with any after-baseline efficacy and/or safety data were included in the respective analysis set. Continuous variables were described by mean, median, standard deviation (SD), minimum as well as maximum, and categorical variables by absolute numbers and percentages. Interferential analyses were performed as primary comparison for categorical endpoints evaluated in a by-visit manner. For categorical nonbinary endpoints, separate van Elteren test stratified by baseline status was applied per visit or overall, whichever was applicable. If approximation of the chi-square distribution was not given, Fisher’s exact test was used for testing without adjustment for baseline status. For continuous endpoints collected over time, a mixed-model repeated measurement was used with considered endpoint as dependent variable, baseline as covariate and treatment, and visit and time multiplied by visit as fixed effects. Subgroup analyses by antigen type (IAV or IBV, IAV alone, IBV alone, ADV alone, combination influenza virus/adenovirus, and others) were performed for selected efficacy parameters as described in Results.

### Metabolite screening during pharmacokinetic study.

To investigate the metabolization of enisamium iodide in humans, plasma samples were obtained from healthy participants in a phase I clinical trial aimed to investigate the influence of food intake on the pharmacokinetics of a single dose of enisamium iodide and conducted in the Vienna General Hospital at the Medical University of Vienna, Vienna, in 2009/2010. The study was performed in accordance with the Declaration of Helsinki, ICH-GCP, and Austrian clinical trial law (EudraCT no. 2009-015382-32) and approved by the Ethics Commission of the Medical University Vienna and the General Hospital Vienna (reference no. 868/2009, dated 27 November 2009) after review of the clinical trial protocol, investigator’s brochure, patient information with informed consent, insurance policy, and qualification of the investigator. Patients were recruited from the database of the study site. During the study, 24 healthy volunteers were randomized and assigned to two groups in a crossover design: fasting/fed and fed/fasting. Plasma samples (3 ml, K_3_EDTA Greiner Bio-One tubes) were taken predose (0 h) and following dosing at 0.25, 0.5, 0.75, 1, 1.25, 1.5, 1.75, 2, 2.5, 3, 3.5, 4, 6, 8, 12, 16, 24, and 48 h and stored at –20°C. For the metabolite screening, 12 plasma samples from the fasting group were used.

To perform the metabolite analysis, the fixed volumes of 50 μl (100 μl for predose) for all 12 subjects at different time points were pooled [predose, P1 (0.5 + 1 + 1.5 h), P2 (2 + 2.5 + 3 h), P3 (3.5 + 4 + 6 h), P4 (8 + 12 + 16 h), P5 (24 + 48 h)] samples. Next, 1 ml of each plasma pool was precipitated with 3 ml of ethanol/acetonitrile (50:50, vol/vol) at room temperature. Plasma samples were centrifuged for 20 min at 3,360 × *g* at 8°C and the supernatant was removed. Each precipitate was washed twice with 3 ml of ethanol/acetonitrile (50:50, vol/vol) and centrifuged again for 20 min at 3,360 × *g* at 8°C. The supernatants of each wash step were subsequently combined and centrifuged again to collect any remaining precipitate. The final precipitate was dried under a stream of nitrogen and reconstituted in 100 μl of water/acetonitrile (90:10, vol/vol). A 10-μl aliquot was further diluted with 40 μl of water/acetonitrile (90:10, vol/vol), and 10 μl of this solution was injected onto the high-performance liquid chromatography (HPLC) column.

LC separation of metabolites was achieved on a Thermo Hypersil Keystone HYPERCARB (2.1 by 150 mm, 5 μm) column utilizing a binary gradient of 0.1% formic acid in water (solvent A) and 0.1% formic acid in water/acetonitrile (10:90, vol/vol; solvent B). The gradient schedule was programmed at 95:5 (solvent A:solvent B) for 0 to 1.0 min, 0:100 for 30 min, and 95:5 for 40 min. The LC flow rate was 200 μl/min. Each sample extract was analyzed by reverse-phase liquid chromatography with full-scan high-resolution mass spectrometry detection. Experiments were carried out in full scan (positive ion mode; *m/z* range, 120 to 1,000; scan event 1).

For selected pools, experiments were also carried out in MS/MS mode (scan events 2 to 4). MS^n^ data were recorded in the data-dependent mode at single mass resolution. In the MS^n^ experiments, the instrument operated using an inclusion list of all candidates, detected in full scan. Whenever a compound with the same *m/z* values as the compounds of the inclusion list was detected, the instrument automatically switched to MS^n^ and collected the fragmentation pattern. For the linear ion trap methods, the experiments were carried out in full scan (positive ion mode; *m/z* range, 120 to 1,000; scan event 1) and in MS/MS mode (scan events 2 to 4). For the LTQ-Orbitrap method (accurate mass measurements), the experiments were carried out in full-scan mode (positive ion mode; *m/z* range, 120 to 1000) with a resolution of 60,000 at *m/z* 400.

### Cells, enisamium, and influenza virus infections.

Human epithelial lung carcinoma (A549), human rhabdomyosarcoma (RD), human hepatocellular carcinoma (HepG2), human embryonic kidney (HEK 293T), and Madin-Darby canine kidney (MDCK) cells were purchased from the American Type Culture Collection (ATCC; Manassas, VA). Human epithelial adenocarcinoma (Caco-2) cells were kindly provided by Martin Walsh (Mount Sinai School of Medicine, New York, NY). All cells were cultured at 37°C and 5% CO_2_ in Dulbecco modified Eagle medium (DMEM) supplemented with 10% fetal calf serum (FBS) and 1% penicillin/streptomycin (pen/strep). The Caco-2 and HepG2 cells were grown on collagen-coated plates. Enisamium iodide was synthesized by Farmak and dissolved in DMSO. Favipiravir triphosphate (T-705-TP) was purchased from Santa-Cruz Biotechnology and dissolved in water.

A549 cells were preincubated with various concentrations of enisamium iodide in minimal essential medium (MEM) containing 0.5% fetal calf serum (FCS) at 37°C for 1 h. Cells were next infected with influenza A/WSN/33 (H1N1) virus at a multiplicity of infection (MOI) of 0.01 in MEM containing 0.5% FCS for 1 h. Following infection, the inoculum was removed and replaced with various concentrations of enisamium iodide in MEM containing 0.5% FCS and incubated for 48 h. Plaque assays were performed on MDCK cells in MEM containing 0.5% FCS with a 1% agarose overlay and grown for 48 h at 37°C. Uninfected cells were treated with compounds in parallel for assessment of toxicity using a CellTiter-Blue kit (Promega).

Caco-2, RD, and HepG2 cells were pretreated with enisamium iodide for 6 h prior to infection with influenza A/WSN/33 (H1N1) virus and enisamium iodide was reapplied after infection. Supernatants were collected at 48 h postinfection and titers determined by immunofluorescence staining in 96-well plates as follows. Serial dilutions of the supernatants were made and used to infect monolayers of MDCK cells in a 96-well plate. After 1 h of incubation, the virus was removed and the cells were washed with phosphate-buffered saline (PBS) and then incubated in postinfection medium (DMEM, 0.1% FBS, 0.3% bovine serum albumin [BSA], 1% pen/strep, 1 mg/ml of tosylsulfonyl phenylalanyl chloromethyl ketone [TPCK] trypsin) for 6 h at 37°C. Cells were fixed in 4% paraformaldehyde, washed 3 times in PBS, and permeabilized in 0.5% Triton X-100. Infected cells were stained with a mouse monoclonal antibody against influenza virus NP (HT103, generated at Mount Sinai School of Medicine, New York, NY) and detected with a goat anti-mouse secondary antibody labeled with Alexa Fluor 488 (Invitrogen). The number of infected cells was quantified on an imaging plate cytometer (Celigo; Nexcelom) and used to determine the titer (PFU per milliliter) based on a standard curve from a known-titer virus stock. Uninfected cells were treated with compounds in parallel for assessment of toxicity.

### Measurement of viral RNA levels in a minigenome assay.

HEK 293T cells were transfected with plasmids expressing the subunits of the influenza A/WSN/33 (H1N1) virus RNA polymerase (pcDNA3-PB1, pcDNA3-PB2, and pcDNA3-PA), the viral nucleoprotein (pcDNA3-NP), and a viral RNA template based on segment 5 (NP-encoding genome segment) (14, 23). As a negative control, a PB1 active-site mutant (PB1a) was used in which the active-site SDD motif was mutated to SAA (14). Enisamium iodide was added to the cell culture medium 15 min after transfection. Twenty-four hours after transfection, cells were washed in PBS and the total RNA was extracted using TRIzol and isopropanol precipitation (15). 5S rRNA and IAV vRNA and mRNA steady-state levels were subsequently analyzed by radioactive primer extension and 6% denaturing PAGE as described previously (15, 24). Phosphorimaging was performed on an FLA7000 Typhoon scanner (GE Healthcare). GFP experiments were performed through transfection of pcDNA3-eGFP, and GFP expression was measured using a SpectraMax plate reader.

### Measurement of RNA polymerase activity in a cell-free system.

The subunits of the A/WSN/33 (H1N1) influenza virus RNA polymerase with a TAP tag on PB2 (pcDNA3-PB1, pcDNA3-PB2-TAP, and pcDNA3-PA) were expressed in HEK 293T cells and purified as a heterotrimeric complex using IgG Sepharose chromatography (15, 24). Next, 0.5 mM CTP, 0.5 mM UTP, 0.05 mM ATP, 0.01 mM GTP, 0.5 mM ApG, 0.001 mM [α-^32^P]GTP, T-705 triphosphate, enisamium iodide, and VR17-04 at the desired concentrations, and 0.5 μM 5′ and 3′ vRNA promoter strands were added to the purified RNA polymerase. After incubation at 30°C for 15 min, samples were analyzed by 20% denaturing PAGE and autoradiography (15).

## Supplementary Material

Supplemental file 1
