# Supplementary material for: Enisamium Reduces Influenza Virus Shedding and Improves Patient Recovery by Inhibiting Viral RNA Polymerase Activity
Source: Antimicrob Agents Chemother. 2021 Mar 18;65(4):e02605-20. doi: 10.1128/AAC.02605-20 (PMC8097484; doi:10.1128/AAC.02605-20)
Supplement: Supplemental file 1 [file AAC.02605-20-s0002.pdf]

Supplemental information for:

Enisamium reduces influenza virus shedding and improves patient recovery by inhibiting viral RNA polymerase activity

Aartjan J.W. te Velthuis[2,9,#,$], Tatiana G. Zubkova[1,9], Megan Shaw[3*], Andrew Mehle[4], David Boltz[5], Norbert Gmeinwieser[6], Holger Stammer[6], Jens Milde[6], Lutz Müller[7#], Victor Margitich[8]

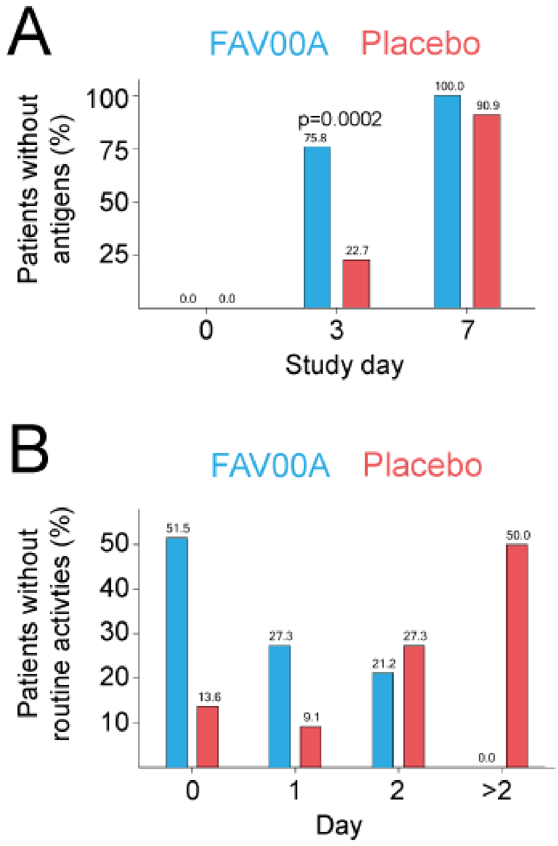

Figure S1. Enisamium treatment reduces viral antigen levels and improves patient activity the subgroup of patients with influenza infection. A) Patients in whom influenza virus antigens were not detected by immunofluorescence staining of nasal swabs (%). B) Patients without routine activities (%). P values were determined by Fisher's exact test.

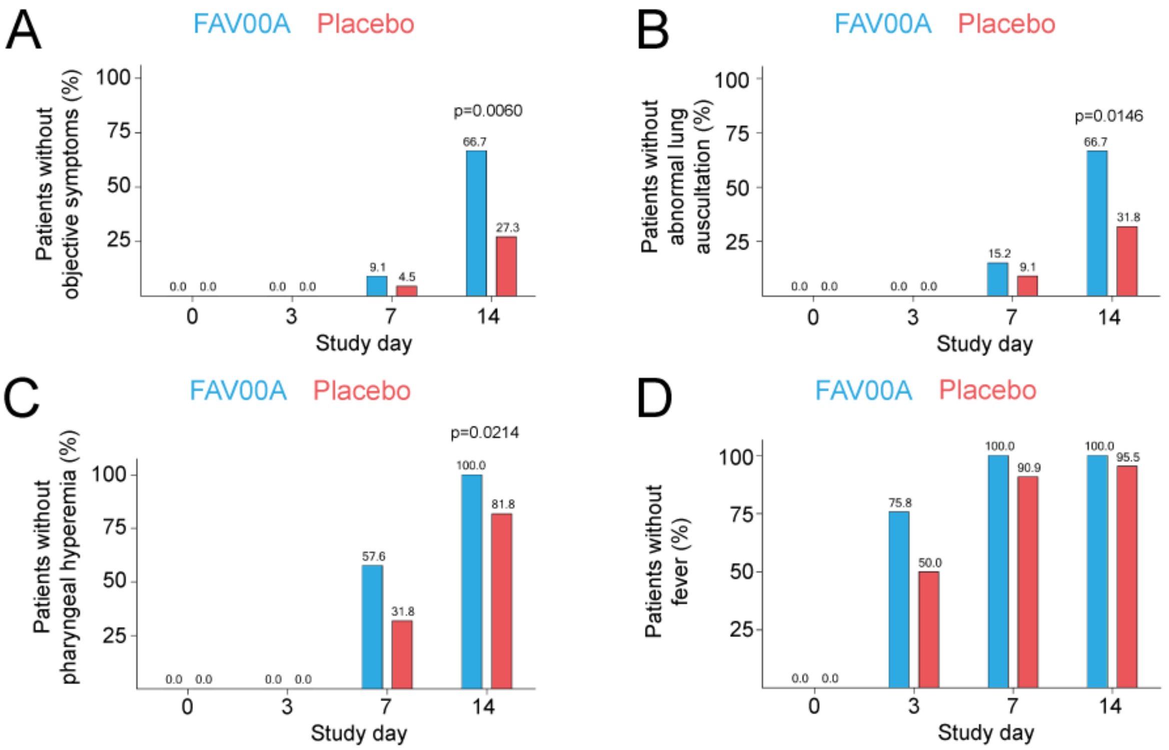

Figure S2. Enisamium treatment reduces objective symptoms in the subgroup of patients with influenza. A) Patients without objective symptoms at different visit days (%). B) Patients without abnormal breath sounds at different visit days (%). C) Patients without pharyngeal hyperemia at different visit days (%). D) Patients without fever at different visit days (%). P values were determined by Fisher's exact test.

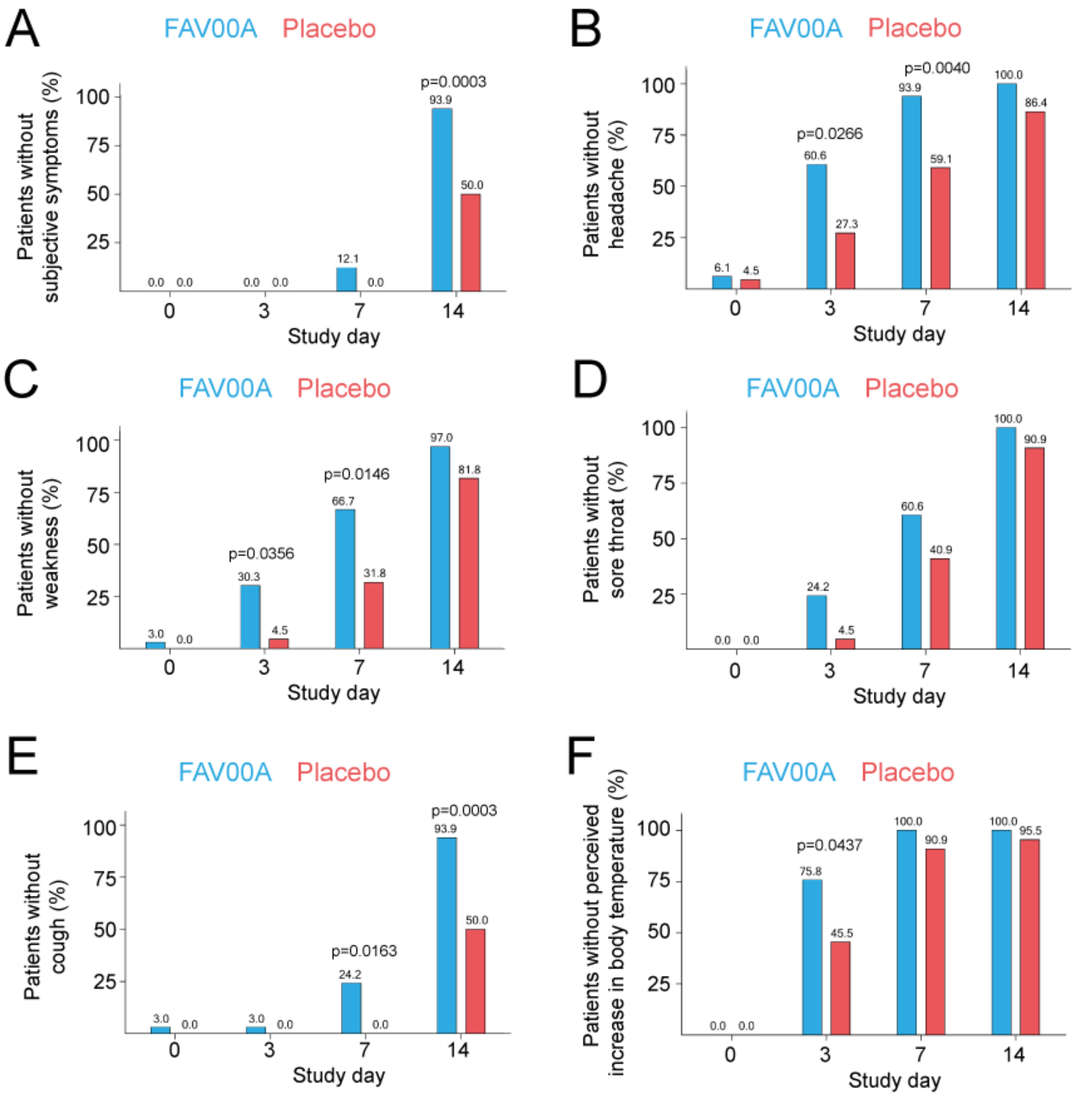

Figure S3. Enisamium treatment reduces subjective symptoms in the subgroup of patients with influenza infection. A) Patients without subjective symptoms at different visit days (%). B) Patients without at different visit days (%). C) Patients without weakness at different visit days (%). D) Patients without sore throat at different visit days (%). F) Patients without cough at different visit days (%). E) Patients without elevated body temperature at different visit days (%). P values were determined by Fisher's exact test.

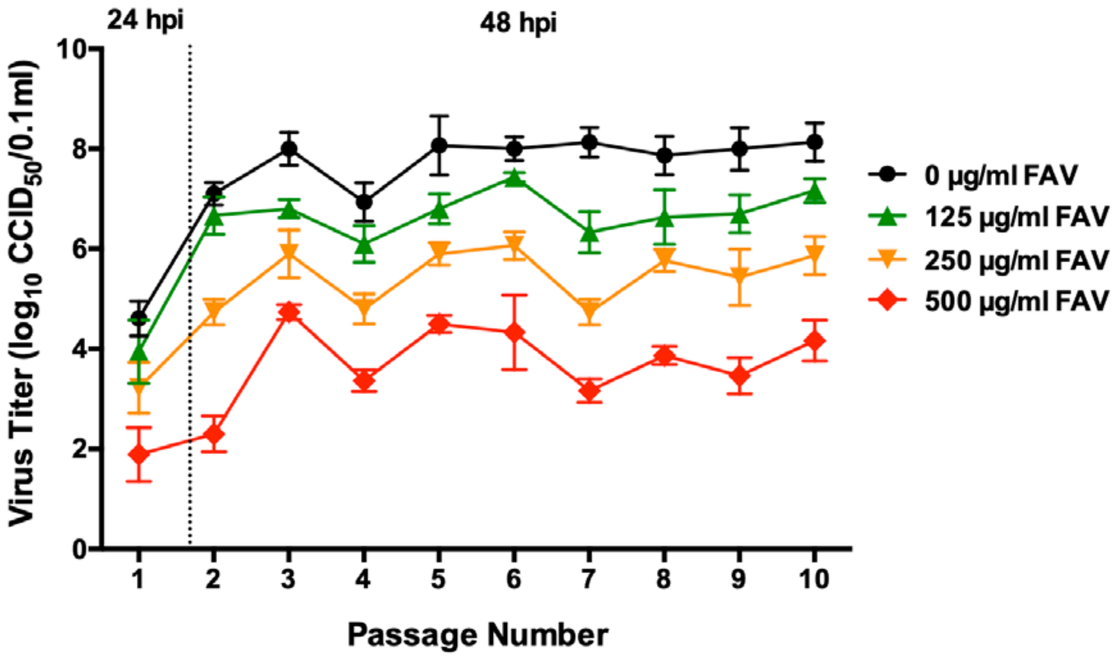

Figure S4. Effect of FAV00B on influenza A/Brisbane/59/2007 (H1N1) virus replication in NHBE cells after 1 to 10 passages of virus in cells treated with enisamium. NHBE cells were infected and treated with enisamium at four concentrations (0, 125, 250, and 500 µg/ml). Twenty-four hours post infection (hpi, Passage 1) and 48 hpi (Passages 2-10), respectively, supernatants were harvested and the resulting virus titers were determined on MDCK cells. Virus was grown under low drug pressure (125 µg/ml in passages 1 and 2) and moderate drug pressure (250 µg/ml in passages 3-10) using virus grown in the preceding passage in NHBE cells. At 0 µg/ml, the virus was serially passaged in the absence of enisamium using the preceding virus passage. Statistical comparisons within each column, comparing rows (simple effect within columns) by two-way ANOVA indicated no statistically significant differences between virus titers at passage 3 compared to passage 10 for each of the lines that was analyzed. Each line (representing infected cells treated with a different concentration of enisamium) was significantly different from all the other lines ($P < 0.0001$).
